# Association between Arsenic Exposure from Drinking Water and Plasma Levels of Soluble Cell Adhesion Molecules

**DOI:** 10.1289/ehp.10277

**Published:** 2007-07-17

**Authors:** Yu Chen, Regina M. Santella, Muhammad G. Kibriya, Qiao Wang, Maya Kappil, Wendy J. Verret, Joseph H. Graziano, Habibul Ahsan

**Affiliations:** 1 Departments of Environmental Medicine and Medicine, and New York University Cancer Institute, New York University School of Medicine, New York, New York, USA; 2 Department of Environmental Health Sciences, Mailman School of Public Health, Columbia University, New York, New York, USA; 3 Department of Health Studies and Cancer Research Center, University of Chicago, Chicago, Illinois, USA; 4 School of Public Health, University of California, Berkeley, California, USA; 5 Department of Epidemiology, Mailman School of Public Health, Columbia University, New York, New York, USA

**Keywords:** arsenic, Bangladesh, cardiovascular disease, epidemiology, environmental epidemiology, endothelial dysfunction, vascular inflammation

## Abstract

**Background:**

Epidemiologic studies of cardiovascular disease risk factors and appropriate biomarkers in populations exposed to a wide range of arsenic levels are a public health research priority.

**Objective:**

We investigated the relationship between inorganic arsenic exposure from drinking water and plasma levels of soluble intercellular adhesion molecule-1 (sICAM-1) and soluble vascular adhesion molecule-1 (sVCAM-1), both markers of endothelial dysfunction and vascular inflammation, in an arsenic-exposed population in Araihazar, Bangladesh.

**Methods:**

The study participants included 115 individuals with arsenic-related skin lesions participating in a 2 × 2 randomized, placebo-controlled, double-blind trial of vitamin E and selenium supplementation. Arsenic exposure status and plasma levels of sICAM-1 and sVCAM-1 were assessed at baseline and after 6 months of follow-up.

**Results:**

Baseline well arsenic, a long-term measure of arsenic exposure, was positively associated with baseline levels of both sICAM-1 and sVCAM-1 and with changes in the two markers over time. At baseline, for every 1-μg/L increase in well arsenic there was an increase of 0.10 ng/mL [95% confidence interval (CI), 0.00–0.20] and 0.33 ng/mL (95% CI, 0.15–0.51) in plasma sICAM-1 and sVCAM-1, respectively. Every 1-μg/L increase in well arsenic was associated with a rise of 0.11 ng/mL (95% CI, 0.01–0.22) and 0.17 ng/mL (95% CI, 0.00–0.35) in sICAM-1 and sVCAM-1 from baseline to follow-up, respectively, in spite of recent changes in urinary arsenic as well as vitamin E and selenium supplementation during the study period.

**Conclusions:**

The findings indicate an effect of chronic arsenic exposure from drinking water on vascular inflammation that persists over time and also suggest a potential mechanism underlying the association between arsenic exposure and cardiovascular disease.

Inorganic arsenic is a natural element of the earth crust. It enters drinking water supplies from natural deposits or from agricultural and industrial practices. Long-term exposure to arsenic in groundwater has been related to elevated risks of cancer of the bladder, lungs, skin, kidneys, and liver (Chen CJ et al. 1988; Chen CL et al. 2004; Morales et al. 2000; Tseng WP 1989); diabetes (Tseng CH et al. 2002; Wang SL et al. 2003); cardiovascular disease (CVD) (Chen CJ et al. 1995, 1996; Chiou et al. 1997; Tseng CH et al. 2003; Wang CH et al. 2002); adverse pregnancy outcomes (Ahmad et al. 2001; von Ehrenstein et al. 2006); and a decrease in children’s intellectual function (von Ehrenstein et al. 2007; Wasserman et al. 2004).

Evidence of high-level arsenic exposure (> 200 μg/L) on vascular disease is largely based on a series of epidemiologic studies in southwestern Taiwan (Ch’i and Blackwell 1968; Chen CJ et al. 1996; Chiou et al. 1997; Tseng CH et al. 2003; Wang CH et al. 2002), collectively suggesting that arsenic exposure induces atherosclerosis, the most common pathologic process underlying CVD that often manifests clinically as coronary disease, stroke, or peripheral arterial disease. However, neither a biological mechanism nor the effect of low-level exposure is clear. More recently, in a cross-sectional analysis, we found a positive association between arsenic exposure and high pulse pressure (Chen Y et al. 2006a), an indicator of arterial stiffness that is associated with an increased risk of atherosclerosis (Dart and Kingwell 2001; Safar et al. 2003). Prospective studies of CVD risk factors, biomarkers, and clinical end points in populations exposed to a wide range of arsenic levels are therefore a public health research priority.

Experimental studies have suggested that arsenic increases the production of reactive oxygen species such as hydrogen peroxide (Barchowsky et al. 1999; Chen YC et al. 1998) and hydroxyl radicals (Wang TS et al. 1996), which may then lead to oxidative stress. In turn, arsenic-induced oxidative stress may mediate gene expression, inflammatory responses, or impaired nitric oxide homeostasis (Simeonova and Luster 2004). These events may ultimately lead to endothelial dysfunction, which disrupts the balance in vasomotor tone between relaxation and contraction and increases the risk for vascular diseases such as hypertension and atherosclerosis (Kumagai and Pi 2004).

Circulating markers of systemic inflammation and endothelial dysfunction, such as soluble intercellular adhesion molecule-1 (sICAM-1) and soluble vascular adhesion molecule-1 (sVCAM-1) have been shown to predict future CVD (Blankenberg et al. 2001; Hwang et al. 1997; Ridker et al. 1998, 2000). Expression of ICAM-1 and VCAM-1 in human umbilical vein endothelial cells was higher in cells stimulated with arsenic than in those without arsenic (Hou et al. 2005). Treatment of mice with arsenic trioxide was associated with a clear increase in expression of ICAM-1 and VCAM-1 (Griffin et al. 2000). However, epidemiologic studies are needed to examine these associations in human populations exposed to arsenic at doses relevant to environmental levels of arsenic exposure.

We evaluated the effects of arsenic exposure on plasma levels of sICAM-1 and sVCAM-1 among 115 individuals participating in a 2 × 2 randomized, placebo-controlled, double-blind trial of vitamin E and selenium supplementation in Araihazar, Bangladesh. Because of the availability of plasma and urine samples at baseline and at follow-up, we were also able to evaluate the effects of changes in arsenic exposure, as well as the effects of vitamin E and selenium supplementation, on changes in plasma sICAM-1 and sVCAM-1.

## Methods

### Study participants

The study population consists of individuals with arsenic-related skin lesions who participated in a randomized, double-blind placebo-controlled trial of vitamin E and selenium. The trial was primarily designed to evaluate the effects of vitamin E and selenium in improving skin lesions. Details of the study have been presented elsewhere (Verret et al. 2005). Briefly, a total of 124 participants with arsenic-related skin lesions were recruited from the Health Effects of Arsenic Longitudinal Study (HEALS), a prospective cohort study with 11,746 participants in Araihazar, Bangladesh (Ahsan et al. 2006a). They were randomized to one of four treatment arms: vitamin E (400 mg racemic α-tocopherol), selenium (200 μg l-seleno-methionine), vitamin E and selenium (combination), or placebo. Eligibility criteria included participation in the parent HEALS, 20–65 years of age, and clinical signs of arsenic-induced skin lesions on at least 10% of the body surface area. The first 124 subjects who fulfilled the eligibility criteria and gave oral informed consent were recruited. The study was approved by the Columbia University Institutional Review Board and the Bangladesh Medical Research Council Ethical Committee.

At baseline, participants underwent a full physical examination. Study medication was taken once daily with water, at the same time of day for 6 months (26 weeks). The field research assistants visited each participant every 2 weeks to resupply study medication, assess compliance, and record any adverse events. At the end of the 6-month treatment period, study subjects were again visited by the study physician for a follow-up visit at which all baseline study procedures were repeated.

A blood and a spot urine sample were taken at baseline and at the end of the treatment period. Whole venous blood samples were collected in 10-mL EDTA Vacutainer tubes. A spot urine sample was collected in 50-mL acid-washed tubes. Both blood and urine samples were kept in portable 4°C coolers immediately after collection and were processed within 2–8 hr at the end of the day in the study office located in Dhaka city. The blood samples were spun in a table-top centrifuge to separate the cells and plasma and stored at –80°C until shipment to Columbia University on dry ice within 1–2 months. Upon receipt, both urine and blood samples were stored at –80°C until analysis.

The present analysis included 115 participants who provided blood and urine samples at both baseline and follow-up.

### Skin lesion diagnosis

Arsenic-related skin lesions have a short latency period and may appear within a few years of exposure. The typical natural progression of the disease starts with hyperpigmentation of the skin, known as “melanosis,” followed by (or in parallel with) a characteristic bilateral thickening of the palms and soles known as “hyperkeratosis,” which often includes nodular protrusions. Trained physicians completed a comprehensive physical examination at baseline and follow-up visits. Physicians were blind to information on the arsenic level in participants’ drinking wells. Details of the clinical examination protocol for skin lesion diagnosis have been described previously (Ahsan et al. 2006a). We instituted a structured protocol adapting the method for quantitative assessment of the extent of body surface involvement in burn patients. The present analysis included 115 confirmed cases of skin lesions; 72 participants had only melanosis, and the remaining 43 had both hyperkeratosis and melanosis.

### Arsenic exposure measurements

Total urinary arsenic concentration was measured by graphite furnace atomic absorption spectrometry using a Perkin-Elmer AAnalyst 600 graphite furnace system (PerkinElmer, Wellesley, MA) in the Columbia University Trace Metals Core Laboratory, as described previously (Nixon et al. 1991). Urinary creatinine levels were analyzed by a colorimetric Sigma Diagnostics Kit (Sigma, St. Louis, MO) as described by Slot (1965). We also calculated changes in urinary arsenic from baseline to follow-up. The concentration of total arsenic in urine has previously been shown to be an excellent biomarker of arsenic exposure in this cohort (Hall et al. 2006). Therefore, we consider changes in urinary arsenic concentration to be a good measure of changes in arsenic exposure over time. After the baseline recruitment of the parent cohort study, an arsenic mitigation program including health education, well labeling, and installations of deep wells was initiated as previously described (Chen Y et al. 2007). These efforts have led to an increase of well switching and changes in urinary arsenic among some individuals (Chen Y et al. 2007).

Well arsenic concentration was tested as part of the parent HEALS study. Detailed methods have been described elsewhere (Ahsan et al. 2006a, 2006b; van Geen et al. 2002). Briefly, water samples from all 5,966 tube wells in the study area were collected in 50-mL acid-washed tubes after the well was pumped for 5 min (van Geen et al. 2002). Water arsenic concentrations were analyzed by graphite furnace atomic-absorption spectrometry with a Hitachi Z-8200 system (Hitachi, Tokyo, Japan) at the Lamont-Doherty Earth Observatory of Columbia University (van Geen et al. 2002). Detailed information on well-use history was also collected as part of the parent cohort study at baseline (Ahsan et al. 2006a). On average, participants in the present study had consumed water from the baseline well for 9.1 years. Therefore, we consider our assessment of well arsenic concentration at baseline to be a good measure of long-term historical measure of arsenic exposure.

### Plasma levels of sICAM-1 and sVCAM-1

It has been suggested that levels of sICAM-1 and sVCAM-1 are largely unaffected by transport conditions and are reproducible within subjects over time (Flower et al. 2000; Pai et al. 2002), and that serum and EDTA-plasma samples give comparable results (Flower et al. 2000). The intraclass correlations for samples with time-to-processing up to 36 hr are > 0.75 for sICAM-1, and > 0.60 for sVCAM-1 (Pai et al. 2002). Plasma levels of sICAM-1 and sVCAM-1 were determined by means of enzyme-linked immunosorbent assays using commercial kits (R&D Systems, Minneapolis, MN). The minimum detectable levels for sICAM-1 and sVCAM-1 are 0.35 and 0.60 ng/mL, respectively. The maximum intraassay and interassay precision, expressed as coefficient of variation (CV%), are 4.8 and 10, and 3.6 and 7.8, for sICAM-1 and sVCAM-1, respectively. In epidemiologic studies of biomarkers, a CV < 5% is considered ideal, whereas CVs up to 15% are often acceptable (Tworoger and Hankinson 2006b). Baseline and follow-up samples from the same participants were placed in the same assay batches to avoid interassay variability (Tworoger and Hankinson 2006a, 2006b). Arsenic exposure status and treatment assignments of vitamin E and selenium were masked to laboratory personnel.

### Statistical analysis

We first conducted descriptive analyses to compare distributions of demographic, lifestyle, and arsenic exposure-related variables among participants in quartiles of well arsenic concentration. Multiple linear regression models were conducted to evaluate *a*) the cross-sectional associations of baseline arsenic exposure and levels of sICAM-1 and sVCAM-1; and *b*) the associations of baseline arsenic exposure, changes in arsenic exposure, and antioxidant treatments with changes in levels of sICAM-1 and sVCAM-1 from baseline to follow-up.

In cross-sectional analyses, baseline well arsenic and urinary arsenic were entered in the models alternatively, and we adjusted for age, sex, body mass index (BMI), and smoking status that may be predictors of levels of sICAM-1 and sVCAM-1 (Blankenberg et al. 2001; Hwang et al. 1997; Ridker et al. 1998, 2000). Previous studies from our group have suggested that age, sex, BMI, and tobacco smoking may modify health effects of arsenic toxicity (Ahsan et al. 2006a; Chen Y et al. 2006b). For the associations between baseline arsenic exposure and changes in sICAM-1 and sVCAM-1, baseline urinary and well arsenic were entered in the model alternatively, and we additionally adjusted for changes in urinary arsenic and baseline level of sICAM-1 or sVCAM-1, respectively. We also controlled for treatment assignment in the models because the study was a randomized trial of vitamin E and selenium.

For the associations with treatment regimen, we adjusted for age, sex, BMI, baseline well arsenic, changes in urinary arsenic, and baseline levels of sVCAM-1 or sICAM-1. We also evaluated whether the severity of skin lesions was related to baseline levels and changes in levels of sVCAM-1 or sICAM-1. Participants with melanosis only were compared with those with both melanosis and hyperkeratosis, controlling for the same above-mentioned variables.

Arsenic exposure variables were categorized based on quartiles. We computed least squares means of sICAM-1 and sVCAM-1 according to the categories. In addition, arsenic exposure variables were entered in the models as continuous variables. Models were also run with log-transformed sICAM-1 and sVCAM-1; the results were similar and are therefore not shown. All statistical analyses were performed using the SAS 9.1.3 statistical package for Windows (SAS Institute Inc., Cary, NC).

## Results

The study population in general had a low educational level and consisted of mostly middle-aged men (Table 1). A total of 48.7% of the participants were current smokers. Well arsenic was not associated with duration of well use, sex, smoking status, age, BMI, educational attainment, treatment duration, treatment assignment, urinary arsenic at follow-up, or changes in urinary arsenic. Similar to what was observed in the parent cohort study (Ahsan et al. 2006a; Chen Y et al. 2007), well arsenic was positively related to baseline urinary arsenic (*p* < 0.01) and switching to alternative wells (*p* < 0.02). In addition, the proportion of participants with both melanosis and hyperkeratosis, a later stage of skin lesion, differed according to well arsenic levels (*p* < 0.01). In univariate analyses, well arsenic was positively related to plasma levels of sVCAM-1 at both baseline and follow-up, and to plasma levels of sICAM-1 at follow-up but not at baseline.

The cross-sectional associations of arsenic exposure with levels of sVCAM-1 and sICAM-1 at baseline are presented in Table 2. Baseline well arsenic was positively associated with both baseline sVCAM-1 and sICAM-1 levels and more strongly with sVCAM-1 (*p* for trend < 0.01), with increasing values in increasing well arsenic quartiles. Every 1-μg/L increase in baseline well arsenic was associated with an increase of 0.10 ng/mL (95% CI, 0.00–0.20) and 0.33 ng/mL (95% CI, 0.15–0.51) in sICAM-1 and sVCAM-1, respectively, at baseline. The associations remained significant with additional adjustment for skin lesion stages [0.10 (95% CI, 0.00–0.20) and 0.28 (95% CI, 0.09–0.46), respectively]. Baseline urinary arsenic was also positively associated with sICAM-1 and sVCAM-1 at baseline; however, only the association with sICAM-1 was significant (*p* for trend < 0.01). Every 1-μg/g creatinine increase in baseline urinary arsenic was associated with an increase of 0.10 ng/mL (95% CI, 0.03–0.17) in sICAM-1 at baseline. The association remained similar with additional adjustment for skin lesion stages [0.10 (95% CI, 0.03–0.16)].

Baseline well arsenic was positively related to changes in plasma levels in sICAM-1 and sVCAM-1, adjusting for age, sex, BMI, smoking status, changes in urinary arsenic, and baseline levels of sICAM-1 and sVCAM-1, respectively (Table 3). Every 1-μg/L increase in baseline well arsenic was associated with a rise of 0.11 ng/mL (95% CI, 0.01–0.22) and 0.17 ng/mL (95% CI, 0.00–0.35) in sICAM-1 and sVCAM-1, respectively, from baseline to follow-up. These associations also remained similar after additional adjustment for skin lesion stages [0.09 (95% CI, 0.01–0.20) and 0.15 (95% CI, 0.00–0.32), respectively]. The positive associations appear to be driven by the increases of sICAM-1 and sVCAM-1 in persons with high baseline well arsenic. Among those in the top quartile of baseline well arsenic, we observed an increase of 55.5 and 97.1 ng/mL in sICAM-1 and sVCAM-1, respectively, from baseline to follow-up. Similarly, baseline urinary arsenic was positively related to changes in sICAM-1 and sVCAM-1. Every 1-μg/g creatinine increase in baseline urinary arsenic was associated with a rise of 0.11 ng/mL (95% CI, 0.04–0.18) and 0.12 ng/mL (95% CI, 0.00–0.24) in sICAM-1 and sVCAM-1, respectively, from baseline to follow-up. Again, these associations remained similar with additional adjustment for skin lesion stages [0.12 (95% CI, 0.05–0.19) and 0.11 (95% CI, 0.00–0.23)]. The association between baseline urinary arsenic and the increase in sVCAM-1 appears to be dose response, with greater increases of plasma sVCAM-1 observed at higher quartiles of urinary arsenic. The higher three quartiles of urinary arsenic were associated with a similar increase in plasma sICAM-1. Stages of skin lesions were not associated with the baseline levels of sVCAM-1 and sICAM-1. Changes in urinary arsenic, on the other hand, were not associated with changes in sICAM-1 (Table 3). Although the top quartile of change in urinary arsenic, in which urinary arsenic increased by 124.5 μg/g creatinine, was associated with a greater increase in sVCAM-1 than in other quartiles, the overall trend was not significant. Treatment with selenium and vitamin E, whether considered independently or jointly in the analysis, was not related to changes in either sVCAM-1 or sICAM-1. Stages of skin lesions were not related to changes in levels of sVCAM-1 and sICAM-1.

## Discussion

We observed positive associations of well arsenic concentration, a long-term arsenic exposure measure in the present study, with plasma levels of sVCAM-1 and sICAM-1. The association was consistent in both cross-sectional and longitudinal analyses. However, short-term changes in arsenic exposure (defined as changes in urinary arsenic) and short-term treatment with selenium and vitamin E over 6 months were not associated with changes in plasma levels of sVCAM-1 and sICAM-1 during the same period. To our knowledge, the present study is the first epidemiologic study that evaluates the associations between arsenic exposure and circulating levels of sVCAM-1 and sICAM-1.

sICAM-1, and sVCAM-1 are markers of vascular inflammation and endothelial dysfunction that are detectable in the circulation. ICAM-1 is a member of the immunoglobulin protein superfamily that mediates cell–cell adhesion (Heiska et al. 1998). Adhesion of circulating leukocytes to the endothelial cell and subsequent transendothelial migration is an important step in the initiation of atherosclerosis (Ross 1993). In part, this process is mediated by cellular adhesion molecules (Adams and Shaw 1994; Cybulsky and Gimbrone 1991), which are expressed on the endothelial membrane in response to inflammatory stimuli. Several large prospective epidemiologic studies have found a significant positive association between increasing serum or plasma concentration of sICAM-1 and risks of total cardiovascular events (Ridker et al. 2000), myocardial infarction (Ridker et al. 1998), and carotid artery atherosclerosis (Hwang et al. 1997). More recently, sICAM-1 has also been associated with a risk of diabetes in prospective cohort studies (Meigs et al. 2004; Song et al. 2007). Development of CVD is the principal complication in type 2 diabetes (Fuller et al. 1983; Jeerakathil et al. 2007; Kannel and McGee 1979). Our finding of the positive association between arsenic exposure and sICAM-1 supports that diabetes and CVD share common antecedents that may be affected by arsenic exposure.

VCAM-1 binds to very late antigen-4, an integrin expressed by monocytes, lymphocytes, and eosinophils; this interaction promotes firm cell–cell adhesion and eventual transmigration of inflammatory cells (Cybulsky and Gimbrone 1991). However, plasma levels of sVCAM-1 have not been reported to be predictive of future CVD risk among apparently healthy individuals (de Lemos et al. 2000; Hwang et al. 1997). In contrast, in persons with previous documented coronary artery disease, elevated plasma sVCAM-1 was associated with future death from CVD, independent of all other inflammatory and soluble adhesion markers (Blankenberg et al. 2001). ICAM-1 is expressed by many cells of hemotopoietic lineage and fibroblasts and thus may be a less specific marker than VCAM-1, which is mainly expressed on atherosclerotic plaques by activated endothelial cells and smooth muscle cells (Blake and Ridker 2002). The positive associations of well arsenic with baseline and changes in plasma sVCAM-1 and sICAM-1 suggest a potential pathway underlying the effect of long-term arsenic exposure on CVD.

In the present study, changes in urinary arsenic over 6 months were not related to changes in sVCAM-1 and sICAM-1. Similarly, treatment with selenium and vitamin E during the same period did not modulate levels of sVCAM-1 and sICAM-1, although experimental studies have suggested that selenium and vitamin E treatment may inhibit the expression of VCAM-1 and ICAM-1 (Koga et al. 2004; Zhang et al. 2002). Our findings on the effect of vitamin E supplementation are consistent with short-term trials in smokers, postmenopausal women, and patients with diabetes that did not find significant effect of vitamin E supplementation alone on these markers (Koh et al. 1999; Tousoulis et al. 2003; Upritchard et al. 2000). On the other hand, high levels of baseline well arsenic and baseline urinary arsenic, which were significantly correlated with baseline well arsenic, were related to increases in sVCAM-1 and sICAM-1 over time. In the present study, participants had consumed water from the baseline wells for an average of 9 years. Taken together, the findings suggest that the effect of high-level, long-term arsenic exposure on vascular inflammation and endothelial dysfunction persists over time in spite of short-term changes in exposure level and antioxidant treatment. In a prospective case–cohort study of risk of arsenic-related skin lesions nested in the same parent cohort study, we also observed that high levels of baseline well arsenic were predictive of incidence of arsenic-related skin lesions, regardless of changes in exposure level over 2 years (Hall et al. 2006). Additional studies are needed to assess whether long-term changes in arsenic exposure and long-term selenium and vitamin E supplementation could influence subsequent changes in levels of sVCAM-1 and sICAM-1 and risk of other health effects related to arsenic exposure.

The findings of the present study should be interpreted with caution. First, the study population consists of mostly men and smokers with skin lesions. The results may not be generalizable to nonsmokers, women, or persons with no skin lesions. The literature and our previous analyses have suggested that men and cigarette smokers are more susceptible to health effects of arsenic exposure (Ahsan et al. 2006b; Bates et al. 2004; Chen CL et al. 2004; Chen Y et al. 2006b; Ferreccio et al. 2000; Karagas et al. 2004). To the extent that susceptibility to the effects of arsenic exposure on levels of sVCAM-1 and sICAM-1 is associated with smoking and male sex, the observed associations may differ from those in the overall population in the study area. Cutaneous abnormalities are well-known early signs of chronic arsenic poisoning and may lead to arsenic-related skin cancer. However, no studies have suggested that persons with skin lesions are more or less likely to develop CVD, apart from the fact that they may have a higher level of arsenic exposure. The observed positive association between arsenic exposure and sVCAM-1 and sICAM-1 is not likely a consequence of skin lesions because there was no association between the severity of skin lesions and plasma levels of sVCAM-1 and sICAM-1. Additional adjustment for skin lesion stages in the models did not change the effect estimates appreciably. Second, because the sample size was small and the arsenic exposure levels in persons with skin lesions were high, we were not able to conduct detailed analyses to evaluate effects of arsenic exposure < 100 μg/L. Future large studies are required to study the associations of low-level arsenic exposure with markers of vascular inflammation and endothelial dysfunction in healthy persons.

Strengths of this study include the availability of multiple arsenic exposure measures to study long-term and recent changes of exposure, the prospective nature of the longitudinal analysis, the random assignment of antioxidant treatments, and the large variation in exposure level in the study population. In summary, we found positive associations of well arsenic concentration with plasma levels of sVCAM-1 and sICAM-1 in persons with skin lesions, suggesting the effect of chronic arsenic exposure on inflammation and endothelial dysfunction. Future studies are needed to confirm the associations in other populations.

## Figures and Tables

**Table 1:** Distributions of demographic, lifestyle, and antioxidant treatment assignments according to well arsenic concentrations in the study population.

		Baseline well arsenic concentration levels in quartiles				
	Overall	Q1 (3–96 μg/L)	Q2 (97–230 μg/L)	Q3 (231–381 μg/L)	Q4 (382–864 μg/L)	*p*-Value^a^
Total no.	115	29	29	29	28	
Age in years (mean ± SD)	46.8 ± 9.0	48.9 ± 9.5	48.7 ± 8.2	45.3 ± 8.2	44.4 ± 9.4	0.12
BMI (mean ± SD)	18.9 ± 2.5	18.9 ± 2.5	19.3 ± 2.9	18.8 ± 2.2	18.8 ± 2.5	0.85
Education in years (mean ± SD)	2.4 ± 3.3	2.3 ± 3.2	2.3 ± 2.9	2.0 ± 3.3	3.1 ± 3.9	0.59
Male (%)	88.7	89.7	89.7	89.7	85.7	0.95
Smoking status (%)						
Past smokers	26.1	20.7	17.2	27.6	39.3	
Current smokers	48.7	62.1	58.6	41.4	32.1	0.25
Treatment assignment (%)						
Placebo	25.2	24.1	24.1	24.1	28.6	0.98
Vitamin E only	25.2	27.6	17.3	27.6	28.6	
Selenium only	24.4	24.1	27.6	20.7	25.0	
Vitamin E and selenium	25.2	24.1	31.0	27.6	17.9	
Skin lesion types (%)						
Melanosis only	62.6	68.9	82.8	62.1	35.7	< 0.01
Hyperkeratosis and melanosis	37.4	31.0	17.2	37.9	64.3	
Treatment duration in weeks (mean ± SD)	26.5 ± 0.6	26.5 ± 0.5	26.6 ± 0.6	26.4 ± 0.6	26.6 ± 0.6	0.48
Well use duration of baseline well (mean ± SD)	9.1 ± 6.2	9.3 ± 5.8	7.6 ± 4.6	10.0 ± 6.0	9.7 ± 8.1	0.46
Switched to other wells (%)	49.6	34.5	37.9	55.2	71.4	0.02
Baseline urinary arsenic (μg/g creatinine)	336.4 ± 313.8	263.2 ± 213.2	266.3 ± 173.8	450.5 ± 430.1	366.8 ± 345.7	0.05
Follow-up urinary arsenic (μg/g creatinine)	263.1 ± 348.5	207.8 ± 144.3	183.8 ± 149.8	298.5 ± 317.4	365.9 ± 584.2	0.17
Changes in urinary arsenic (μg/g creatinine)	–73.3 ± 291.4	–55.4 ± 101.8	–82.5 ± 1119.5	–151.9 ± 179.5	–0.93 ± 535.8	0.26
Plasma levels (ng/mL)						
sICAM-1 at baseline	397.5 ± 110.7	399.2 ± 95.3	401.1 ± 136.8	389.7 ± 110.3	399.9 ± 101.3	0.41
sICAM-1 at follow-up	403.3 ± 152.1	401.8 ± 91.3	387.4 ± 130.6	378.7 ± 112.4	446.7 ± 235.2	0.04
sVCAM-1 at baseline	636.9 ± 201.1	597.4 ± 162.3	603.5 ± 165.7	640.6 ± 215.4	708.7 ± 241.9	< 0.01
sVCAM-1 at follow-up	638.4 ± 243.8	601.9 ± 192.6	567.7 ± 162.7	618.7 ± 203.8	769.6 ± 342.0	< 0.01

a *p*-Value was based on chi square or ANOVA.

**Table 2:** Associations of arsenic exposure variables and skin lesion types with baseline plasma levels of sVCAM-1 and sICAM-1.

			sVCAM-1 (ng/mL)			sICAM-1 (ng/mL)		
Variables	Mean^a^	No.	Adj mean/regression coefficient^b^,^c^	SD	*p*-Value	Adj mean/regression coefficient^b^,^c^	SD	*p*-Value
Baseline well arsenic (μg/L)								
3–96	52.7	29	644.4	45.6		411.2	25.1	
97–230	158.2	29	667.4	46.8		423.4	25.8	
231–381	320.2	29	745.4	51.5		429.3	28.4	
382–864	566.5	28	788.1	48.1		443.3	26.5	
Per μg/L^c^		115	0.33	0.09	< 0.01	0.10	0.05	0.04
Baseline urinary arsenic (μg/g creatinine)								
51–135	100.5	28	715.9	50.6		394.8	26.5	
136–211	172.1	29	644.1	48.1		410.8	25.2	
212–448	297.4	30	706.4	48.3		419.6	25.3	
449–1,126	784.3	28	752.5	49.5		469.5	25.9	
Per μg/g creatinine^c^		115	0.04	0.07	0.53	0.10	0.04	< 0.01
Baseline skin lesion types								
Melanosis only		72	681.1	39.9		417.7	21.86	
Hyperkeratosis and melanosis		43	733.1	43.6	0.15	426.0	23.90	0.89

Adj, adjusted.

a Category-specific mean values of the arsenic variables in the left column.

b Means of sVCAM-1 and sICAM-1 for arsenic exposure categories were adjusted for sex, age, BMI, and smoking status; means for skin lesion categories were adjusted for sex, age, BMI, smoking status, and baseline well arsenic.

c Regression coefficients and *p*-values were computed with the arsenic exposure variable entered as a continuous variable in the model.

**Table 3:** Associations of arsenic exposure variables, skin lesion types, and antioxidant treatments with changes in plasma levels of sVCAM-1 and sICAM-1 from baseline to follow-up.

			Changes from baseline to follow-up					
			sVCAM-1 (ng/mL)			sICAM-1 (ng/mL)		
	Mean^a^	No.	Adj mean/regression coefficient^b^,^c^	SD	*p*-Value	Adj mean/regression coefficient^b^,^c^	SD	*p*-Value
Baseline well arsenic (μg/L)								
3–96	52.7	29	18.3	41.5		15.4	26.5	
97–230	158.2	29	–21.0	43.8		–9.9	28.1	
231–381	320.2	29	26.2	48.6		0.9	30.5	
382–864	566.5	28	97.1	46.0		55.5	28.3	
Per μg/L^c^		115	0.17	0.09	0.05	0.11	0.05	0.03
Baseline urinary arsenic (μg/g creatinine)								
51–135	100.5	28	–3.5	48.0		8.3	30.3	
136–211	172.1	29	16.3	44.7		22.7	28.4	
212–448	297.4	30	48.1	44.5		20.3	28.0	
449–1,126	784.3	28	68.0	61.3		19.5	38.2	
Per μg/g creatinine^c^		115	0.12	0.06	0.05	0.11	0.04	< 0.01
Changes in urinary arsenic (μg/g creatinine)								
–1,062 to –145	–314.6	29	19.4	19.4		31.9	26.5	
–144 to –41	–80.7	28	20.5	20.5		2.4	29.0	
–40 to –4	–22.6	29	25.6	25.6		9.6	29.6	
–3 to 288	124.5	29	55.1	55.1		18.1	29.4	
Per μg/g creatinine^c^		115	–0.04	0.06	0.56	–0.03	0.06	0.12
Baseline skin lesion types								
Melanosis only		72	12.6	35.3		10.7	22.50	
Hyperkeratosis and melanosis		43	64.2	40.1	0.13	28.9	25.28	0.30
Treatment assignment								
No selenium		57	18.0	38.9		2.5	24.5	
Selenium		58	42.3	35.3	0.49	28.4	21.9	0.24
No vitamin E		58	48.6	39.3		11.2	24.6	
Vitamin E		57	11.7	35.3	0.30	19.7	22.2	0.71
Placebo		29	23.4	46.5		–2.2	29.4	
Selenium only		28	80.2	46.4		24.9	29.4	
Vitamin E only		29	16.2	44.8		7.3	28.5	
Vitamin E and selenium		29	7.6	41.3	0.49	32.0	26.0	0.68

Adj, adjusted.

a Category-specific mean values of the arsenic variables in the left column.

b Means of sVCAM-1 and sICAM-1 for arsenic exposure categories were adjusted for sex, age, BMI, smoking status, treatment regimen, and baseline levels of sVCAM-1 (for changes in sVCAM-1) or sICAM-1 (for changes in sICAM-1); adjusted means for baseline urinary or well arsenic categories were also adjusted for changes in urinary arsenic. Adjusted means for treatment regimen and skin lesion categories were adjusted for sex, age, BMI, smoking status, baseline well arsenic, changes in urinary arsenic, and baseline levels of sVCAM-1 (for changes in sVCAM-1) or sICAM-1 (for changes in sICAM-1).

c Regression coefficients and *p*-values were computed with the arsenic exposure variable entered as a continuous variable in the model.
